# A Rare Case of Isolated Left Ventricular Non-compaction in an Elderly Patient

**DOI:** 10.7759/cureus.2886

**Published:** 2018-06-26

**Authors:** Jai D Parekh, John Iguidbashian, Shweta Kukrety, Kelsey Guerins, Paul G Millner, Venkata Andukuri

**Affiliations:** 1 Internal Medicine, Creighton University Medical Center, Omaha, USA; 2 Creighton University Medical Center, Omaha, USA; 3 Pediatrics, Creighton University Medical Center, Omaha, USA

**Keywords:** cardiomyopathy, heart failure, non-compaction, ventricular dysfunction, reduced ejection fraction, cardiology, cardiac imaging, electrophysiology

## Abstract

A 61-year-old male, with a history of emphysema, obstructive sleep apnea, and hypertension, presented to the emergency room with worsening shortness of breath over a three-month period. The patient also complained of orthopnea, paroxysmal nocturnal dyspnea, and progressively worsening lower limb swelling. On examination, the patient had jugular venous distension, bilateral lower extremity edema, and bibasilar crackles. The laboratory evaluation showed an elevated B-natriuretic peptide level and a normal troponin level. A transthoracic echocardiogram (TTE) showed a reduced left ventricular ejection fraction (LVEF) of 20%-25% with prominent hyper-trabeculations noted in the left ventricle, most prominent in the lateral and apical walls. These findings were concerning for left ventricular non-compaction (LVNC). The patient underwent left heart catheterization, which did not show obstructive coronary disease as a cause of his cardiomyopathy. The patient was managed with guideline-directed therapy for heart failure and was started on warfarin due to the increased risk of thromboembolism associated with LVNC. During his admission, he exhibited multiple episodes of nonsustained ventricular tachycardia and was subsequently evaluated by electrophysiology (EP). He was discharged home with a wearable cardioverter defibrillator with instructions to follow up with EP in three months for an evaluation of implantable cardioverter-defibrillator (ICD) placement for primary prevention.

## Introduction

Left ventricular non-compaction (LVNC) is a rare cardiomyopathy that is being diagnosed with increasing frequency as awareness of the disorder spreads and diagnostic modalities advance. The etiology is thought to occur in utero, as physiologic sinusoids fail to fully compact into mature myocardium [1]. The result is residual myocardial trabeculations extending into the ventricle, forming deep intra-trabecular recesses that impair cardiac function. Clinical presentation is highly variable, ranging from asymptomatic to debilitating heart disease or even arrhythmia and cardiac arrest. The majority of patients are asymptomatic into adulthood, where they are incidentally diagnosed on cardiac imaging. LVNC has also been reported to have an association with congenital heart defects and will present in infancy or early childhood with signs of heart failure, such as failure to thrive, lack of development, or arrhythmia. It is rare, however, to develop symptoms from isolated LVNC later in adulthood, as seen in our patient.

## Case presentation

**Figure 1:**
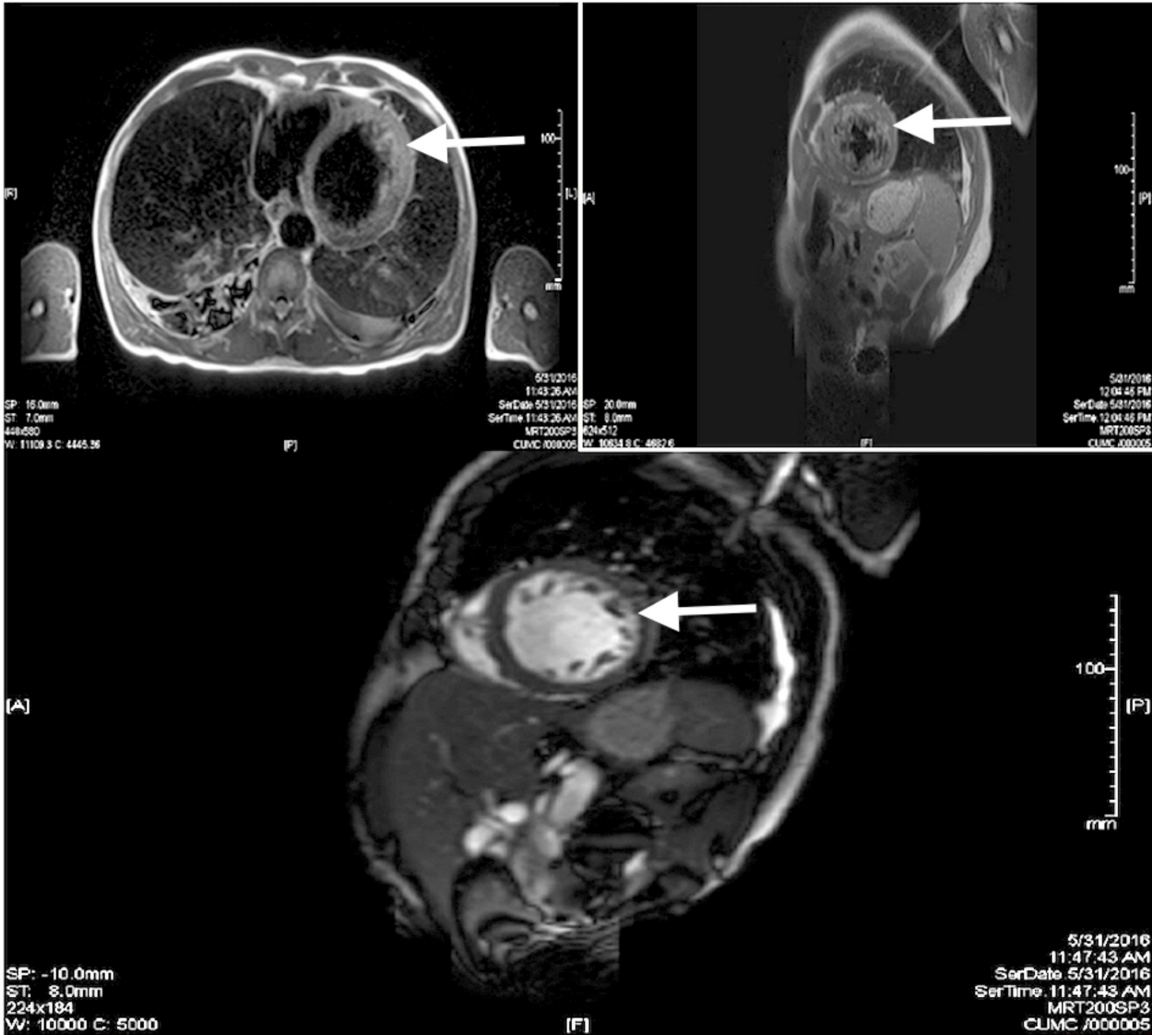
Cardiac MRI showing hyper-trabeculations (white arrows), assisting in the diagnosis of LVNC MRI: magnetic resonance imaging

A 61-year-old male, with a history of emphysema, obstructive sleep apnea, and hypertension, presented to the emergency room with worsening shortness of breath over a three-month period. The patient also complained of orthopnea, paroxysmal nocturnal dyspnea, and progressively worsening lower limb edema. On examination, the patient had jugular venous distension, bilateral lower extremity edema, and bibasilar crackles. The laboratory evaluation showed a B-natriuretic peptide level of 11,065 pg/ml and a troponin level of < 0.04 ng/ml. A transthoracic echocardiogram showed a reduced left ventricular ejection fraction (LVEF) of 20%-25% with prominent hyper-trabeculations noted in the left ventricle, most prominent in the lateral and apical walls. These findings were concerning for LVNC. Cardiac magnetic resonance imaging (CMRI) showed a non-compacted to compacted myocardium ratio of 5:1 at the left ventricular apex (Figure 1), confirming the diagnosis of LVNC. The patient underwent left heart catheterization, which did not show obstructive coronary disease as an etiology for the cardiomyopathy. The patient was managed with guideline-directed therapy for heart failure, including carvedilol, losartan, furosemide, hydralazine, and isosorbide mononitrate. He was also started on warfarin due to the increased risk of thromboembolism associated with LVNC. He had episodes of non-sustained ventricular tachycardia during his admission and was subsequently evaluated by electrophysiology (EP). He was discharged home with a wearable cardioverter defibrillator with instructions to follow up with EP in three months for an evaluation of implantable cardioverter defibrillator (ICD) placement for primary prevention.

## Discussion

LVNC is a rare cardiac condition characterized by excessively prominent left ventricular trabeculae and deep intertrabecular recesses, with continuity between the left ventricular cavity and these recesses. Isolated LVNC occurs in the absence of other congenital abnormalities. Typically, the left ventricle is involved, although the involvement of the right ventricle has also been described [2]. It is believed to occur due to an intrauterine arrest of compaction of the myocardial fibers during embryogenesis [1,3]. Patients typically present with heart failure, atrial or ventricular arrhythmias, sudden cardiac death, or thromboembolism. Diagnosis is usually made by echocardiography with color Doppler. Jenni et al. proposed the following criteria for diagnosis: maximal end-systolic ratio of non-compacted to compacted myocardium > 2:1 in the parasternal short axis view, the predominant presence of the trabecular meshwork in the apical or mid-inferior and lateral wall, and color Doppler evidence of flow into the inter-trabecular recesses from the left ventricular cavity [4]. CMRI can also be used for diagnosis when a maximum end-diastolic non-compacted to compacted myocardium ratio of > 2.3 is found. Recently, cardiac computed tomography has also been used for the diagnosis of LVNC, thus enabling the simultaneous evaluation of complex intra-cardiac pathology and ventricular function as well as for coronary artery morphology with a single test [5].

The systolic dysfunction in LVNC is thought to occur due to a relative ischemia of the myocardium with a mismatch of myocardial oxygen supply and demand. The multiple prominent trabeculations cause a restriction in filling, an abnormal ventricular relaxation pattern and diastolic dysfunction, with a generally poor eventual outcome for patients [6]. Two unique complications of LVNC are the increased risk of both fatal arrhythmias as well as the risk of thromboembolism. There is no specific therapy for LVNC; management is based on clinical manifestations. Patients presenting with heart failure are treated with guideline-directed medical therapy. Patients with end-stage heart failure are candidates for heart transplantation. In LVNC, the broader use of implantable cardioverter defibrillator (ICD) therapy should be considered in addition to patients with LVEF < 35% or in patients who have survived an episode of sustained ventricular tachycardia or sudden cardiac arrest. Anticoagulation should be considered in patients with LVEF < 40%. The prognosis of symptomatic patients is usually poor. Further research is needed to provide clear guidelines for the treatment of the above complications.

## Conclusions

In conclusion, LVNC is a diagnosis that should be considered for patients presenting with signs and symptoms of heart failure and abnormal structural findings on various cardiac imaging modalities. Although primarily diagnosed in childhood, identifying and diagnosing LVNC in adults has significant clinical implications. With early identification, potentially preventable complications, such as ventricular arrhythmias and thromboembolism, may be avoided. In addition, patients need to be started on appropriate therapeutic management for heart failure symptoms to prevent progression.
